# Non-viral delivery of CRISPR–Cas9 complexes for targeted gene editing via a polymer delivery system

**DOI:** 10.1038/s41434-021-00282-6

**Published:** 2021-08-06

**Authors:** Jonathan O’Keeffe Ahern, Irene Lara-Sáez, Dezhong Zhou, Rodolfo Murillas, Jose Bonafont, Ángeles Mencía, Marta García, Darío Manzanares, Jennifer Lynch, Ruth Foley, Qian Xu, A Sigen, Fernando Larcher, Wenxin Wang

**Affiliations:** 1 Charles Institute of Dermatology, University College Dublin, Dublin, Republic of Ireland; 2 Epithelial Biomedicine Division, Centro de Investigaciones Energéticas Medioambientales y Tecnológicas (CIEMAT), Madrid, Spain; 3 Centro de Investigación Biomédica en Red de Enfermedades Raras (CIBERER) ISCIII, Madrid, Spain; 4 Fundación Instituto de Investigaciones Sanitarias de la Fundación Jimenez Díaz, Madrid, Spain; 5 Department of Bioengineering Universidad Carlos III de Madrid, Madrid, Spain

**Keywords:** Transfection, Genetic vectors, Nanoparticles

## Abstract

Recent advances in molecular biology have led to the CRISPR revolution, but the lack of an efficient and safe delivery system into cells and tissues continues to hinder clinical translation of CRISPR approaches. Polymeric vectors offer an attractive alternative to viruses as delivery vectors due to their large packaging capacity and safety profile. In this paper, we have demonstrated the potential use of a highly branched poly(*β*-amino ester) polymer, HPAE-EB, to enable genomic editing via CRISPRCas9-targeted genomic excision of exon 80 in the COL7A1 gene, through a dual-guide RNA sequence system. The biophysical properties of HPAE-EB were screened in a human embryonic 293 cell line (HEK293), to elucidate optimal conditions for efficient and cytocompatible delivery of a DNA construct encoding Cas9 along with two RNA guides, obtaining 15–20% target genomic excision. When translated to human recessive dystrophic epidermolysis bullosa (RDEB) keratinocytes, transfection efficiency and targeted genomic excision dropped. However, upon delivery of CRISPR–Cas9 as a ribonucleoprotein complex, targeted genomic deletion of exon 80 was increased to over 40%. Our study provides renewed perspective for the further development of polymer delivery systems for application in the gene editing field in general, and specifically for the treatment of RDEB.

## Introduction

Gene therapy has long been heralded as a new breakthrough in modern molecular medicine as it enables the treatment of disorders at the genetic level [1–3]. Delivery strategies of gene therapy relying on viral vectors, whilst efficient, raise safety concerns regarding immunogenicity and insertional mutagenesis [4–7]. However, non-viral vectors such as cationic polymers offer an attractive alternative to viruses given their facile synthesis, versatility and improved safety profile [8–10]. These polymers can condense nucleic acids into nanoparticles via electrostatic interactions to protect them from enzymatic degradation, and facilitate transport across the cell membrane in an efficient and cytocompatible manner [11–17]. For example, poly(*β*-amino esters) (PAEs), and in particular linear PAEs (LPAEs), have been demonstrated to be versatile polymer gene delivery candidates both in vitro and in vivo [18–20]. Although initial results of using LPAEs are very encouraging, further enhancement and functionalization of these polymers is limited owing to their linear configuration. On the other hand, previous investigations have highlighted that branched structures demonstrate significantly enhanced complexation with nucleic acids, as well as containing multiple groups which can be functionalized to assist with cell uptake, and with endosomal escape mediated by high buffering capacity [21–23]. In fact, breakthrough research by Zhou et al. has successfully developed novel highly branched PAE (HPAE) polymers for gene delivery that have outperformed their linear counterparts by orders of magnitude in delivering large DNA constructs into cells [2, 3, 12–14, 24–31].

In the field of translational gene therapy research, therapeutics based on gene editing have been widely adopted as an alternative to classical gene replacement therapy, using genetically engineered site-specific nucleases to correct underlying mutations in inherited disorders [32, 33]. In general, the various gene editing platforms in use function on similar principles, being designed so that engineered non-specific nucleases are attached to sequence-specific DNA binding domains and mediate DNA cleavage. These DNA breaks can then be repaired by either the error-prone non-homologous end joining or in a sequence-specific manner through the introduction of a homologous DNA repair template. Three main gene editing platforms are currently in use: zinc finger nucleases, transcription activator-like effect proteins and the most recently discovered gene editing technology: clustered regulatory interspaced short palindromic repeats (CRISPR) and its CRISPR associated Cas proteins. In particular, the advent of the CRISPR–Cas9 genome editing system has revolutionised the field with its superior simplicity and versatility compared to previous designer nuclease technologies [34–36].

Even with the emergence of CRISPR–Cas9, delivery of nucleic acids into target cells and tissues remains a key bottleneck for clinical translation, owing to safety concerns associated with the use of viruses, and low transfection efficiency observed with non-viral delivery systems [34–36]. Viral vectors continue to be used as the primary delivery system in the CRISPR–Cas9 field; however, non-viral systems are emerging as a promising alternative [37]. One of the benefits of CRISPR–Cas9 technology is that stable integration into the host genome is not necessary to mediate therapeutic levels of correction if delivery efficiency is high [34, 35, 38–42]. Nevertheless, long-term expression represents a major drawback for CRISPR–Cas9 as it increases the likelihood of off-target nuclease activity mediating undesirable cleavage [35, 43]. To date, the research into the use of polymers for delivery of gene editing technologies has been minimal. However, with the potential of CRISPR–Cas9 for clinical translation, there exists a current unmet need for a non-viral system to deliver such a disruptive platform technology.

Gene editing with CRISPR–Cas9 delivered as a ribonucleoprotein (RNP) complex offers several distinct advantages over conventional delivery of nucleic acids (plasmid DNA or mRNA). As the Cas9 and single-guide RNA (sgRNA) enter the cell as a pre-assembled complex, there is no risk of Cas9 causing DNA integration into the host genome, in addition to avoiding problems with gene expression, RNA degradation and protein folding [44, 45]. While native Cas9 has a net positive charge, each sgRNA is extremely anionic and makes the overall complex highly electronegative [46–48]. These properties indicate that in theory, delivery of the protein–RNA complex could be achieved in much the same manner as nucleic acid delivery.

In this study, we successfully demonstrated in vitro gene editing mediated by a HPAE-EB cationic polymer as a delivery vector. As a therapeutic proof of concept, we chose to target the excision of exon 80 of the COL7A1 gene. This site is known as a frequent location of mutations in patients suffering from recessive dystrophic epidermolysis bullosa (RDEB), a subtype of the rare skin fragility disorder family of epidermolysis bullosa. This excision strategy could be extended to other genetic disorders including Duchenne muscular dystrophy, where targeted removal of mutation-containing genomic regions holds great promise for clinical translation [49, 50]. As an ex vivo RDEB treatment, Bonafont et al. designed and validated a dual sgRNA strategy for mediating precise excision of exon 80 in the COL7A1 gene, using electroporation to deliver CRISPR–Cas9 as a RNP to edit skin cells. Corrected bioengineered skin grafted onto mice showed collagen VII restoration in the epidermal basement membrane zone and the formation of mature anchoring fibrils at the dermal-epidermal junction of the gene-edited RDEB skin [51]. In this paper, we explored the potential of delivering this dual-guide CRISPR system as a CRISPR–Cas9 DNA construct (CRISPR-C7) and as a CRISPR–Cas9 RNP complex using a HPAE-EB as a non-viral vector for treatment of RDEB.

## Materials and methods

### Polymer synthesis

The HPAE-EB polymer used in this research work was previously identified as a lead candidate polymer for mediating optimal gene delivery efficiency while maintaining cell viability. Full details regarding polymer design and synthesis have been previously reported [13, 24].

### Plasmid design

The CRISPR-C7 construct was derived from the pX601 plasmid (Addgene, Watertown, MA, USA) and modified based on plasmid pDC315+iresGFP. Guide RNA sequences flanking exon 80 of the COL7A1 gene were designed by CRISPOR (http://crispr.mit.edu/).

### RNP design and complexation

Both crRNAs and tracrRNA were diluted to 100 µM with nuclease-free duplex buffer with HiFi Cas9 nuclease (IDT, Coralville, IA, USA) used as indicated in the guidelines of the manufacturer. RNP complexes were prepared such that sgRNA (crRNA + tracrRNA):Cas9 molar ratio was 6.6:1.

### Polyplex formation

DNA and HPAE-EB were diluted in 25 mM sodium acetate (Sigma Aldrich, St. Louis, MO, USA) across different polymer:DNA or polymer:RNP weight/weight (w/w) ratios, before mixing at a 1:1 volume/volume (v/v) ratio and vortexing for 30 s. DNA–lipid complexes using Lipofectamine 3000 (Sigma Aldrich) were used as a control. Lipofectamine 3000 reagent was mixed at a 1:1 v/v ratio with DNA solution, mixing 0.6 or 0.85 µl Lipofectamine with 0.5 or 1 µg of DNA, respectively, after optimising the protocol as suggested by the manufacturer. The resulting solutions were incubated at room temperature for 10 min to allow polyplex formation. Full methods detailing assessments of polyplexes for biophysical properties complexation, encapsulation, buffering capacity, size, and charge are found in Supplementary Methods.

### Cell culture

Human embryonic 293 kidney cells (HEK293) (CRL-1573, ATCC, Manassas, VA, USA) were cultured in Dulbecco’s Modified Eagle Medium 6429 (Sigma Aldrich) with 10% foetal bovine serum (Thermo Fisher Scientific, Waltham, MA, USA) and 1% penicillin/streptomycin (Thermo Fisher Scientific). Immortalised primary human RDEB keratinocytes were cultured using standard cell culture techniques in keratinocyte growth complete FAD medium (KCa) as described by Bonafont et al. [51], and incubated at 37 °C and 5% CO_2_in a humidified incubator. Cells were prepared 24 h prior to transfection with seeding density per well of 4 × 10^4^ cells for 96-well plates and 1 × 10^5^ for 24-well plates, and transfection was carried out when cells reached 60–70% confluence.

### Cell transfection

HEK293 and RDEB keratinocytes were transfected with HPAE-EB complexed to 0.5 and 1 µg CRISPR-C7 plasmid DNA per well for 96-well plates. Lipofectamine 3000 acted as a commercial transfection reagent control with 0.5 and 1 µg CRISPR-C7 plasmid DNA per well for 96-well plates, as recommended by the supplier. Polyplexes were prepared, mixed with culture medium and added to cells. Medium was replaced after 4 h post transfection with fresh medium. Expression of the reporter gene green fluorescent protein (GFP) was visualised 48 h after transfection with CRISPR-C7 plasmid using an Olympus IX81 fluorescence microscope (Olympus, Tokyo, Japan).

RDEB keratinocytes were transfected with HPAE-EB with 0.5, 2, 4 and 8 µg of RNP per well for 24-well plates at a 20:1 w/w ratio of polymer:RNP.

### Cell viability

Cell viability was assessed 48 h post transfection. Medium was removed and cells were washed with 100 μl of Hank’s Balanced Salt Solution (HBSS) (Sigma Aldrich) per well. One hundred microliters of alamarBlue™ (Thermo Fisher Scientific) working solution (10% alamarBlue™ in HBSS) was added to each well and incubated for 2 h under normal cell culture conditions previously described. Absorbance was recorded at 570 and 600 nm on a SpectraMax M3 multi-plate reader (Molecular Devices, San Jose, CA, USA). Viability calculations are detailed in Supplementary Methods.

### Flow cytometry and fluorescent activated cell sorting (FACS)

To compare transfection efficiency and intensity across different weight ratios and DNA concentrations using the HPAE-EB polymer, flow cytometry analysis was performed using an AcurriC6 (BD Bioscience, Franklin Lakes, NJ, USA). HEK293 cells were transfected in a 96-well plate as previously described, and were prepared for flow cytometry assessment 72 h after transfection. Forward and side scattering were used to gate out cell debris and doublets. GFP fluorescence was detected at 509 nm using a 530/30 nm band pass filter. Propidium iodide (PI) (10 μl per tube) was utilised to identify cells whose membrane had been compromised. At least 10,000 cells were counted per treatment and untransfected cells were used as negative controls for evaluating cellular background autofluorescence. Flow cytometry data were analysed using AccurriC6 software (BD Bioscience). To assess the transfection capability of the optimal HPAE-EB condition on RDEB keratinocytes, FACS was used to identify GFP-positive cells and measure the intensity of fluorescence present. Untransfected RDEB keratinocytes were used as a negative control for the FACS gating strategy. RDEB keratinocytes were transfected and prepared as described for HEK293. A further gate was employed for RDEB keratinocytes to isolate those GFP-positive cells that were deemed highly fluorescent, with sorting done on this population alone. Automated FACS was performed on a BD FACSARIA III cell sorter (BD Biosciences) for identification of overall optimal transfection efficiency in RDEB keratinocytes. Flow cytometry and FACS assessments were carried out in the Flow Cytometry Core Facility at the UCD Conway Institute (Dublin, Ireland).

### DNA isolation and PCR genotyping

Genomic DNA was extracted from whole cell populations using isopropanol precipitation. Five hundred microliters of lysis buffer (Tris [pH 8] 100 mM, EDTA 5 mM, SDS 0.2%, NaCl 200 mM and 1 mg/ml proteinase K; Merck, Kenilworth, NJ, USA) was added directly to each well, and cell lysates were collected in 1.5 ml centrifuge tubes and incubated overnight at 55 °C. DNA was then isolated and resuspended in 1X TE buffer (NEB, Ipswich, MA, USA). NEBNEXT^®^ (M0541) (NEB) was used as the DNA polymerase master mix and PCR was performed with the primers listed in Supplementary Table S1. ImageJ software (NIH, Bethesda, MD, USA) was used for PCR band densitometry calculation.

### Sequence analysis

PCR samples used for editing analysis were sent to Eurofins MWG Operon Inc. (Luxembourg, Luxembourg) for Sanger sequencing and purification. Inference of CRISPR Edits (ICE) analysis (Synthego, Redwood City, CA, USA) was used to confirm editing events and to track formation of insertion–deletion mutations (indels) in a pooled cell population. Chromas software (Technelysium, QLD, Australia) was used to display sequence chromatograms.

### Statistical analysis

All data are represented with the mean ± standard deviation (±SD). For transfection experiments, six technical replicates and three biological replicates were carried out. The rest of the assessments were performed with three technical replicates and three biological replicates, except collagen VII immunocytochemistry where data were collected from two repeats of two independent experiments. Analysis was carried out by a one-way analysis of variance with the posterior Dunnett’s post-hoc multiple comparison test to compare data to control, using the software GraphPad Prism 6.0 (San Diego, CA, USA). *P* values less than 0.05 were considered significant (*p < 0.05, **p < 0.01, ***p < 0.001, ****p < 0.0001). Statistical significance was reported in the figure legends.

## Results

### HPAE-EB polymer efficiently complexes and encapsulates CRISPR-C7 plasmid

**Fig. 1:**
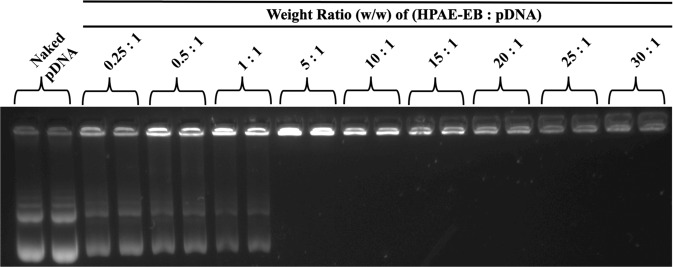
Agarose gel retardation assay to assess the CRISPR-C7 plasmid DNA complexation by the HPAE-EB polymer. The cationic HPAE-EB polymer exhibits significant DNA condensation ability and can retard the movement of anionic DNA through the agarose gel at polymer:DNA w/w ratios ranging from 5:1 to 30:1. At w/w ratios below 5:1, incomplete retarding of DNA is evident as some DNA has migrated down through the gel, indicating insufficient complexation. Naked CRISPR-C7 plasmid alone (without polymer) was used as the control for this experiment. Data are representative of three independent experiments with each w/w ratio assessed in duplicate (*n* = 3).

Inefficient complexation of nucleic acids leads to suboptimal delivery to target cells. The HPAE-EB polymer was assessed for its complexation and encapsulation ability for the CRISPR-C7 plasmid via gel retardation and PicoGreen^®^ assay, respectively (Supplementary Methods). Gel retardation showed that HPAE-EB polymer sufficiently complexed the CRISPR-C7 plasmid, even at a low polymer:DNA w/w ratio of 5:1 (Fig. 1). At higher w/w ratios between 15:1 and 30:1, a marked decrease in DNA signal intensity can be attributed to the strong complexation by HPAE-EB shielding the CRISPR-C7 plasmid and reducing its accessibility for the DNA stain to intercalate with the plasmid.

**Fig. 2:**
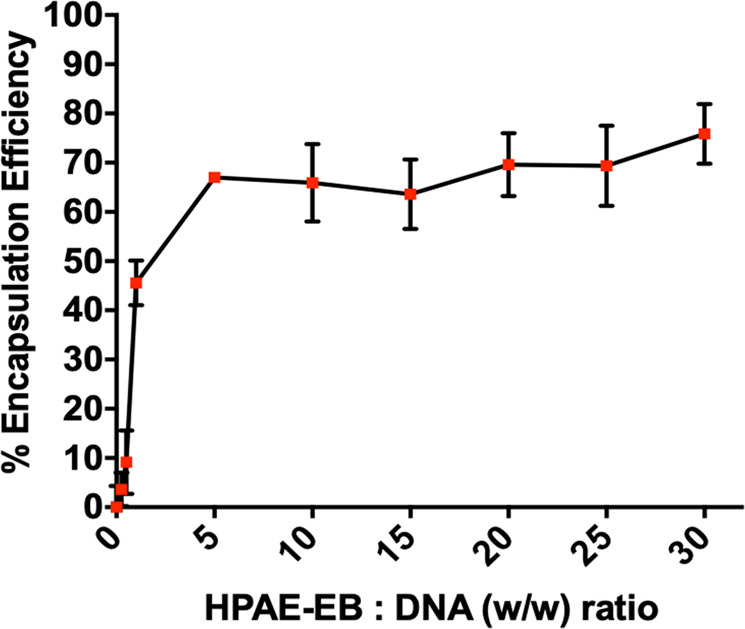
DNA encapsulation efficiency assessment with the HPAE-EB polymer and CRISPR-C7 plasmid complexes. Experiments were performed at polymer:DNA w/w ratios from 0.25:1 to 30:1 using 1 μg of DNA. At low polymer:DNA ratios of 0.25:1 and 0.5:1, the HPAE-EB polymer is incapable of adequately encapsulating the DNA from the PicoGreen^®^ reagent. However, upon reaching w/w ratios of 5:1 and greater, an encapsulation efficiency of ~70% is achieved and maintained, indicating sufficient binding to protect the DNA. Samples were performed in triplicate and presented as average ± standard deviations (SD) for three independent experiments (*n* = 3).

To gain a quantitative understanding of the HPAE-EB’s encapsulation profile, DNA binding affinity across increasing polymer:DNA w/w was examined using the PicoGreen^®^ reagent. Less than 10% encapsulation was achieved at low w/w ratios between 0.25:1 and 1:1, demonstrating inefficient binding of DNA (Fig. 2). In contrast, a dramatic increase was seen at w/w ratios of 2:1 with nearly 50% DNA binding, which further increased to ~70% at w/w ratios of 5:1. Increasing this ratio further to 30:1 only caused a minor improvement in encapsulation, suggesting a plateau is reached for encapsulation of anionic plasmid DNA. These results indicate that the HPAE-EB polymer is a viable delivery vector for encapsulating and shielding the CRISPR-C7 plasmid for gene delivery.

### HPAE-EB exhibits ideal polyplex properties for efficient gene delivery

**Fig. 3:**
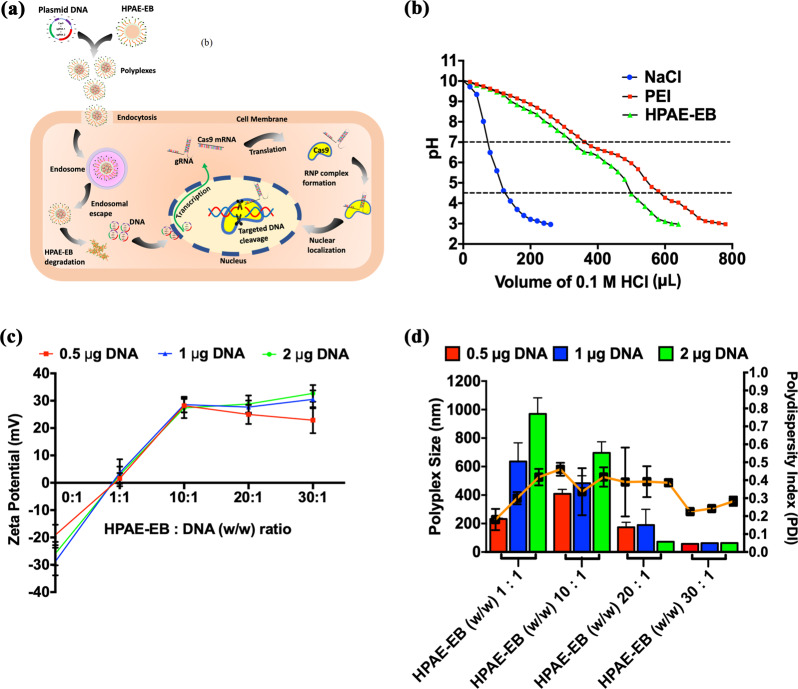
Transfection process and physical characterisation of HPAE-EB polymer alone and complexed to CRISPR-C7 plasmid. **a** Non-viral transfection process using HPAE-EB and CRISPR-C7 plasmid DNA to mediate gene editing. **b** Endosomal buffering capacity of the HPAE-EB polymer. Acid–base titration curve of HPAE-EB, PEI (25 kDa) and NaCl across a wide pH range was performed. PEI (25 kDa) was used as a commercial standard for comparison and NaCl was used as a negative control. With addition of HCl, NaCl shows sharp decline in pH, whereas PEI and HPAE-EB exhibit gradual decrease owing to higher proton buffering capacity (*n* = 3). **c** Zeta potential of HPAE-EB polyplexes with CRISPR-C7 plasmid DNA, complexed at different w/w ratios. DNA alone (0:1 w/w ratio) was used to demonstrate the negative charge of DNA, and from w/w ratios of 10:1 and higher, polyplex surface charge is positive for all DNA quantities. **d** Hydrodynamic size and PDI distribution of the HPAE-EB polymer and CRISPR-C7 plasmid DNA polyplexes at varying DNA quantities and polymer:DNA w/w ratios. As polymer:DNA ratio is increased, there is a sharp decrease in polyplex size across all DNA quantities. PDI distribution is heterogenous throughout all conditions tested. Data are presented as mean ± SD of three replicates from three independent experiments (*n* = 3).

Successful gene delivery is a multifaceted process encompassing several hurdles and barriers, including cellular uptake and endosomal escape (Fig. 3a). To assess the ability of HPAE-EB to navigate these hurdles, endosomal buffering capacity was tracked using an acid–base titration assay (Supplementary Methods). Results showed that HPAE-EB demonstrated high buffering capacity in the pH range 4.5–7 typically found within endosomes and lysosomes (Fig. 3b). The large volume of HCl required to shift the pH of the solution indicates the enhanced capability of HPAE-EB to escape from endosomal compartments. Negative control NaCl displayed negligible buffering capacity, as demonstrated by the sharp decline in pH, while polyethylenimine (PEI), a known polymer with exceptional buffering capacity, exhibited a similar buffering trend to HPAE-EB.

Naked DNA is anionic and displays little to no uptake into cells. However, polyplexes with an overall cationic charge would promote interaction with the cell membrane for cellular uptake. Figure 3c shows that once polymer:DNA w/w ratios reach 10:1 and beyond, HPAE-EB polyplexes display positive surface charges ranging between 20 and 30 mV irrespective of DNA quantity. In contrast, at low w/w ratios of 1:1, HPAE-EB polyplex surface charge is neutral which would be unfavourable for cellular uptake.

Polyplex size is another key determining factor for mediating uptake into cells. In Fig. 3d, at low w/w ratios of 1:1 and 10:1, HPAE-EB polyplexes increase in size relative to overall DNA quantity, reaching nearly 1000 nm in diameter. This points toward incomplete encapsulation and condensation of the CRISPR-C7 plasmid. In contrast, at higher w/w ratios of 20:1 and 30:1, substantially smaller polyplexes of 100–200 nm diameter are consistently detected, providing a size range ideal for facile uptake into cells. Taken together, these results demonstrate that HPAE-EB can form polyplexes of positive surface charge, small size and efficient buffering capacity for mediating efficient gene delivery.

### Transfection efficiency with HPAE-EB: CRISPR-C7 polyplexes in vitro

**Fig. 4:**
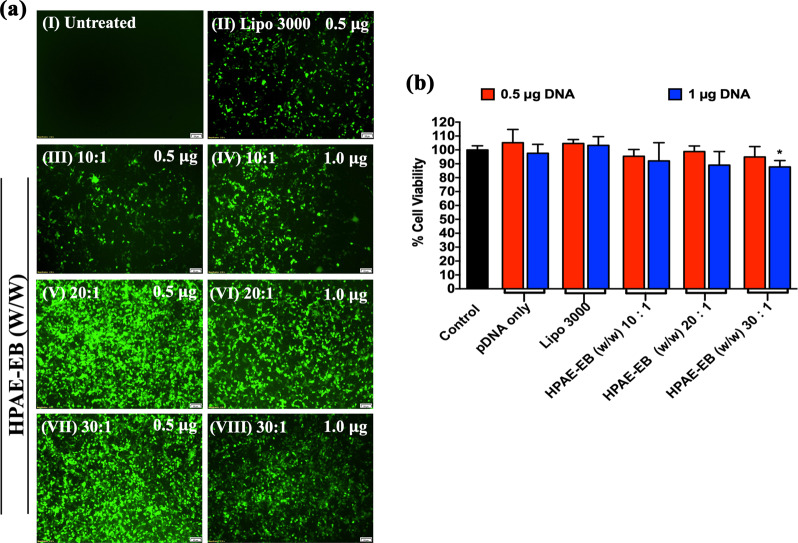
Transfection capability and cytotoxicity of HPAE-EB polymer complexed to CRISPR-C7 plasmid in HEK293 cells. **a** GFP within the CRISPR-C7 plasmid enabled visual transfection in cells. (I) Untreated cells were used as the negative control. (II) Lipofectamine 3000 was used as the commercial reagent for comparison, and treatments with different polymer:DNA w/w ratios and amounts of DNA (III–VIII) were performed. Scale bar 100 µm. Representative images from six replicates of three independent experiments (*n* = 3). **b** Cell viability 72 h post transfection in HEK293 cells by alamarBlue™, where preservation of cell metabolic health post transfections using HPAE-EB polymer and Lipofectamine 3000 was observed. Data were collected from six replicates of three independent experiments and presented as means ± SD (*n* = 3). *p < 0.05, as compared to control values.

High transfection efficiency is crucial for clinical translation of gene therapy. CRISPR-C7 plasmid (containing +iresGFP expression cassette) complexed to HPAE-EB was transfected into HEK293 cells and GFP fluorescence was used to track transfection efficiency qualitatively. Figure 4a demonstrates through fluorescence intensity that HPAE-EB achieves a level of transfection efficiency vastly superior to the commercial transfection reagent Lipofectamine 3000. As transfection efficiency is a key factor in gene editing rates, HPAE-EB:DNA w/w ratios of 20:1 and 30:1 (Fig. 4a, V–VII) would be expected to yield the highest levels of gene editing efficiency.

Next, we sought to investigate the cytotoxic profile of transfection with the CRISPR-C7 plasmid in HEK293 cells by using the alamarBlue^TM^ cell metabolic health assay. HPAE-EB polyplexes were able to maintain over 85% cell viability across increasing w/w polymer:DNA ratios and DNA quantities, comparable to Lipofectamine 3000 which is known to have low levels of cytotoxicity (Fig. 4b). These results were further confirmed by quantification of GFP fluorescence through flow cytometry, after transfecting HEK293 cells with different amounts of DNA and different w/w ratios with the polymer (Supplementary Fig. 1). HPAE-EB at w/w 10:1 achieved a maximum of 5.8% GFP-positive cells. In contrast, HPAE-EB w/w ratios of 20:1 and 30:1 were able to achieve levels of GFP-positive cells ranging from 55 to 67.3%, depending on the DNA quantity, while maintaining overall cell viability around 72% (Supplementary Fig. 1). The increase in PI staining, in particular at lower amounts of DNA, could be explained by the mechanism of cellular uptake of cationic polymers, which involves endocytosis and pore formation in the cell membrane [52, 53]. This pore formation in the cell membrane could facilitate PI uptake into cells and account for the increase in its staining. Nonetheless, cell membrane permeability is also a contributing factor to cellular death and as such should be minimised as much as possible.

**Fig. 5:**
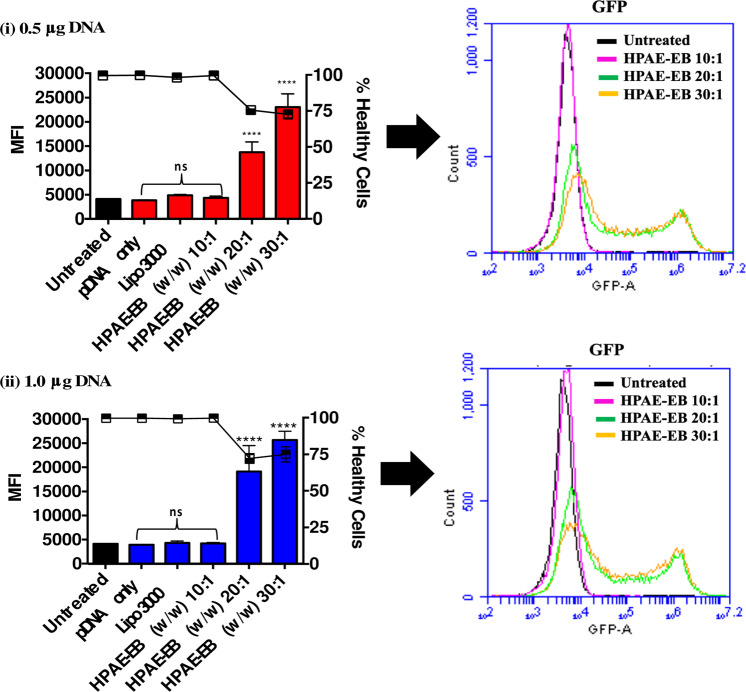
Mean fluorescence intensity (MFI) and cell viability of transfected HEK293 cells. MFI of GFP fluorescence for each transfection condition was grouped into polymer:DNA w/w ratios used and cell viability. In addition, one-plot histogram overlay of GFP fluorescence comparing untreated control and HPAE-EB transfections under different w/w ratios was performed. Only HPAE-EB at w/w ratios of 20:1 and 30:1 yields a statistically significant increase in MFI. All results are shown as mean ± SD, *n* = 3. ****p < 0.0001, as compared to control values.

To quantify fluorescence intensity mediated by the different transfection conditions, the median fluorescence intensity (MFI) for all treatments was recorded and displayed in Fig. 5, this figure also demonstrates that Lipofectamine 3000 and HPAE-EB at w/w 10:1 had MFIs similar to untreated controls. In contrast, transfections with HPAE-EB at w/w ratios of 20:1 and 30:1 resulted in statistically significant increases in MFI, indicating higher numbers of GFP-positive cells with stronger fluorescent intensity. An indirect relationship was observed between cell viability and MFI for transfections with HPAE-EB at w/w of 20:1 and 30:1, consistent with previous flow cytometry gating profiles. Finally, to further demonstrate transfection efficiency achieved by HPAE-EB across a wide range of conditions, overlapped GFP fluorescence intensities were presented on one-plot histograms (Fig. 5). When HPAE-EB:DNA w/w ratios were compared with controls, a clear shift in fluorescence was evident upon increasing w/w ratios across all DNA quantities.

**Fig. 6:**
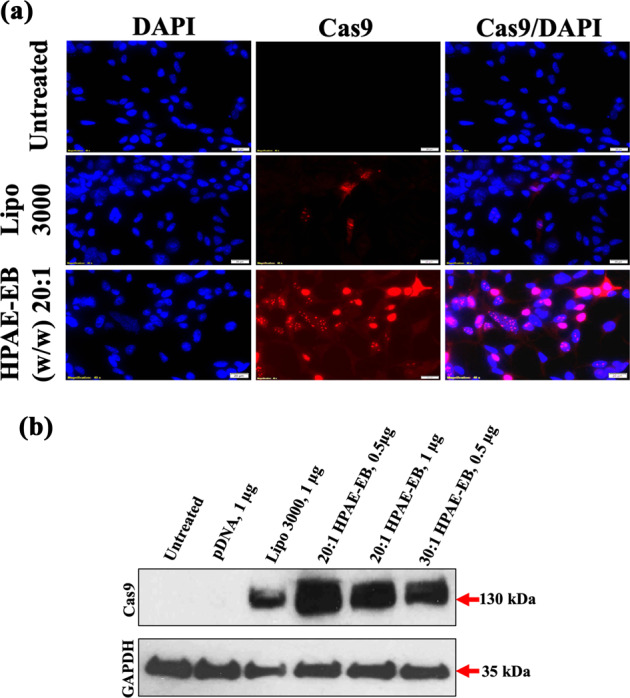
Cas9 production and localisation in transfected HEK293 cells using plasmid CRISPR-C7. **a** Untreated HEK293 cells acted as a negative control and Lipofectamine 3000 was used as the commercial reagent comparison. Samples were fixed 72 h post transfection and stained with Cas9 antibody in situ (red), and with DAPI as the nuclear stain. Magnification at 40×, scale bar 20 µm. **b** Representative western immunoblot for intracellular levels of Cas9 at the optimal conditions from transfections with HPAE-EB compared with the commercial reagent Lipofectamine 3000. Substantial Cas9 production was achieved in HEK293 cells following transfections with HPAE-EB at w/w ratios of 20:1 and 30:1. No expression of Cas9 was present in untreated or pDNA-only treated cells. GAPDH was used as the loading control. Data are representative of three independent experiments (*n* = 3).

To gain further insight into transfection efficiency and CRISPR–Cas9 production, we investigated Cas9 intracellular localisation and production in transfected HEK293 cells (Supplementary Methods). Significant positive Cas9 staining is evident in cells treated with HPAE-EB at a w/w ratio of 20:1 with DNA, predominantly localised around the nucleus, the site of action for CRISPR gene editing (Fig. 6a). Transfection with HPAE-EB mediates higher expression of Cas9 compared to Lipofectamine 3000, in line with previous transfection results. Western immunoblotting for Cas9 supplemented the immunocytochemical results, with high protein expression in cell lysates of HEK293 cells transfected with HPAE-EB polyplexes and harvested after 72 h (Fig. 6b). These results show that HPAE-EB mediates efficient transfection while maintaining cytocompatibility in HEK293 cells, and post transfection, cells express high levels of Cas9 to mediate gene editing.

### Transfection with HPAE-EB polyplexes yields efficient gene editing in vitro in HEK293 cells

**Fig. 7:**
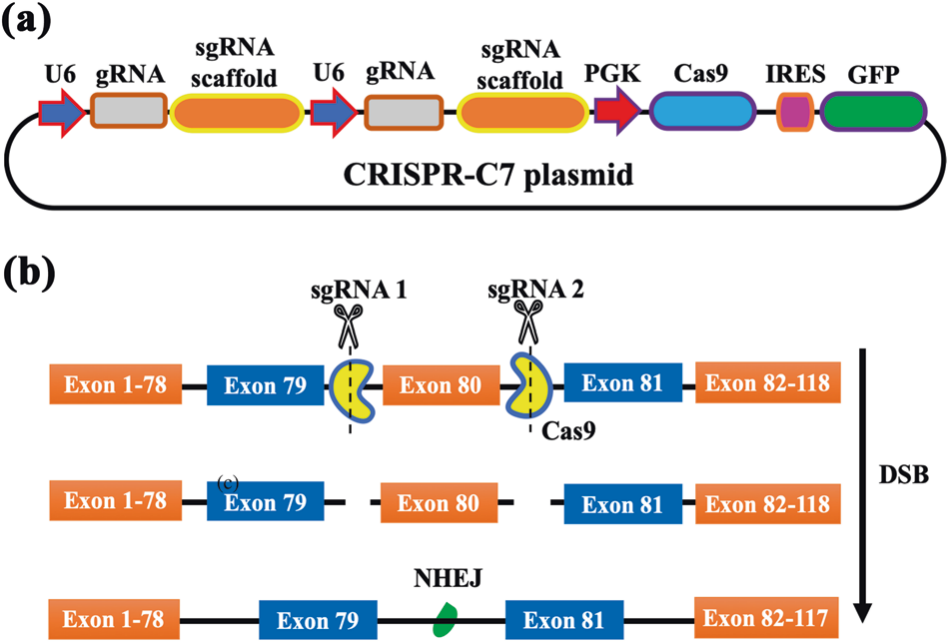
CRISPR–Cas9 gene editing strategy for targeted exonal excision of mutation-containing exons in COL7A1 gene. **a** CRISPR-C7 plasmid schematic detailing construct design containing dual-guide RNA sequences and Cas9 coupled with GFP reporter for transfection efficiency evaluation. **b** Dual-guide RNA strategy for targeted genomic deletion of pathogenic mutation-containing exon 80 in COL7A1 gene.

**Fig. 8:**
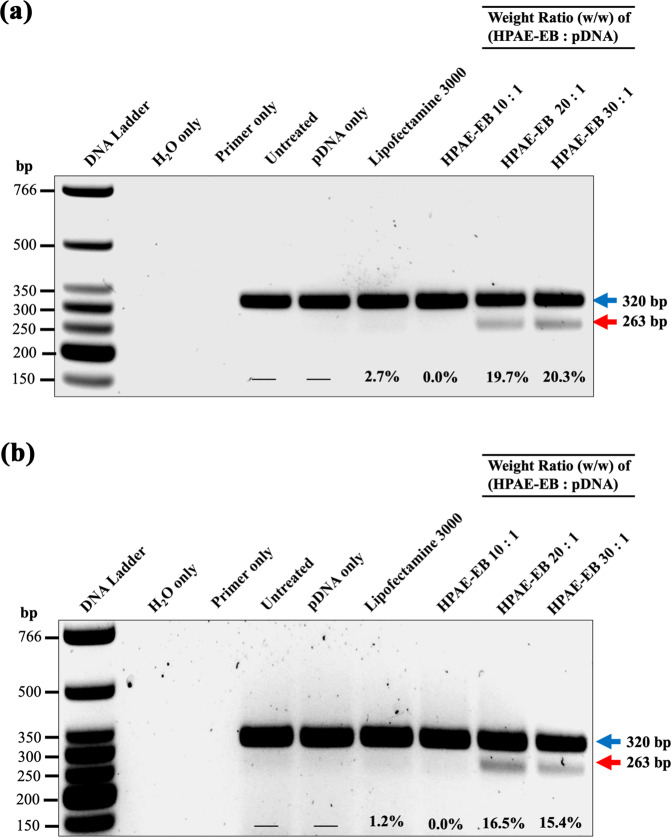
PCR analysis of genomic DNA from HEK293 cells transfected with CRISPR-C7 plasmid under different conditions. HEK293 cells were treated with **a** 0.5 µg and **b** 1 µg of CRISPR-C7 plasmid. Upper arrow at 320 bp represents unedited DNA, while lower arrow at 263 bp represents DNA lacking exon 80. Lipofectamine 3000 was used as the commercial reagent control. Excision efficiency (%) estimated by densitometry analysis showed a maximum editing of 20.3%, achieved using a HPAE-EB:pDNA w/w ratio of 30:1. Data are representative of three independent experiments (*n* = 3).

We developed an all-in-one vector to express Cas9 and a dual-guide RNA sequence targeted to a therapeutically relevant frequent mutation site, for targeted genomic deletion (Fig. 7a). Target cut sites resided within intronic regions flanking exon 80 of COL7A1 gene (Fig. 7b) [51]. In vitro cleavage activity was assessed by PCR amplification of DNA spanning target sites, to determine whether double-stranded breaks (DSBs) induced indels and subsequent excision of exon 80 in transfected HEK293 cells. The expected band pattern was apparent in HEK293 cells transfected with CRISPR-C7 plasmid, but not with control DNA, and demonstrated targeted deletion of 57 bp consistent with the distance between target cut sites, inclusive of exon 80 (Fig. 8). These data confirmed the all-in-one expression plasmid’s functionality in vitro. Densitometry analysis of band intensity showed that HPAE-EB polyplexes at polymer:DNA w/w ratios of 20:1 and 30:1 achieved deletion efficiency between 15 and 20%. In contrast, Lipofectamine 3000 only managed to yield up to 1.2% deletion efficiency. These results complement previous GFP fluorescence and Cas9 protein production transfection experiments, which showed HPAE-EB polyplexes at w/w ratios of 20:1 and 30:1 to be optimal for gene delivery.

**Fig. 9:**
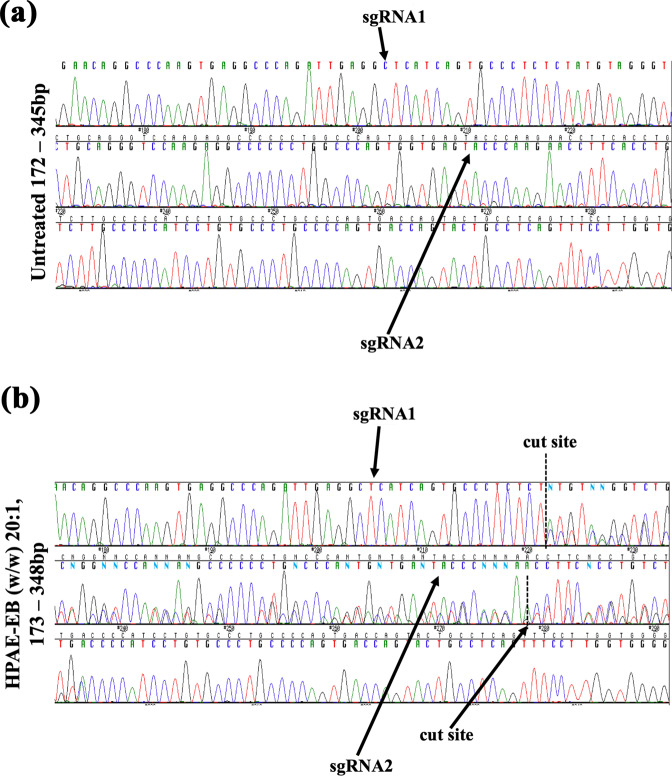
Chromatograms of Sanger sequenced PCR amplicons of COL7A1 gene. Guide RNAs denoting cut sites (dashed lines) are indicated highlighting the dual-guide strategy employed. **a** Sharp single peaks representative of no editing are seen in the control sample. **b** In contrast, overlapping peaks denoting the mixed pool population of cells are seen directly at cut sites confirming indel generation by HPAE-EB:pDNA (w/w) 20:1 using 0.5 µg DNA treated cells. Data are representative of three replicates from two independent experiments (*n* = 2).

PCR amplicons underwent Sanger sequencing and were analysed using ICE software, which infers CRISPR activity from sequence traces, to stratify the indel spectrum generated. Sequence traces show single peaks throughout for control DNA, consistent with no indel formation (Fig. 9a). At guide RNA cut sites, multiple overlapping signal peaks begin, denoting the random indels generated by DSBs mediated by CRISPR–Cas9 activity within transfected HEK293 cells (Fig. 9b). ICE analysis showed that targeting introns flanking exon 80 created indels which resulted in 17% of sequenced amplicons lacking exon 80 of COL7A1 gene, with 15% of indels consisting of a 57 bp deletion. Additional editing events of roughly 2% included larger deletions up to 64 bp encompassing exon 80 (Supplementary Table S2). These results confirm our previous PCR results demonstrating that 15–20% targeted deletion of the 57 bp sequence between the cut sites was achieved. Overall, these data suggest that HPAE-EB can deliver an all-in-one expression vector to efficiently excise target genomic sites for application in gene editing.

### CRISPR–Cas9-based gene editing in an RDEB in vitro model

**Fig. 10:**
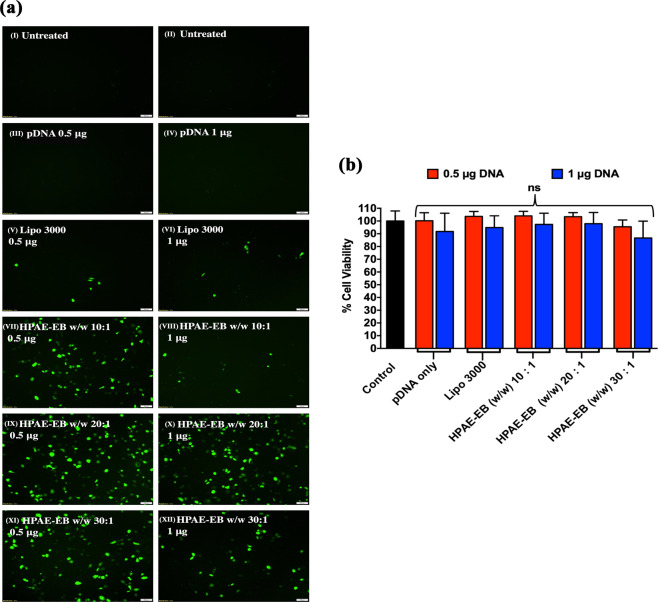
RDEB keratinocytes transfections with the HPAE-EB polymer and CRISPR-C7 plasmid. **a** Efficient transfection was evaluated by GFP expression. Cells were treated with different polymer:DNA ratios and DNA amounts (VII–XII). Furthermore, Lipofectamine 3000 was used as the commercial reagent for comparison (V, VI), untreated cells were used as a negative control (I, II) and plasmid DNA as the vector control (III, IV). Scale bar 100 µm. Representative images from six replicates of three independent experiments (*n* = 3). **b** alamarBlue™ test showed high preservation of RDEB keratinocytes viability 72 h post transfection using different HPAE-EB polymer conditions complexed to DNA, and Lipofectamine 3000 as a control. Data were collected from six replicates of three independent experiments and presented as mean ± SD (*n* = 3).

While Lipofectamine 3000 failed to achieve any significant transfection efficiency in RDEB keratinocytes, the HPAE-EB polymer exhibited far superior transfection efficiencies across a number of conditions, although not to the same level of intensity previously seen in HEK293 cells (Fig. 10a). A general trend was evident in RDEB keratinocytes whereby lower amounts of DNA, 0.5 μg per well, mediated higher transfection efficiency than 1 μg DNA per well across all HPAE-EB w/w ratios of 10:1, 20:1 and 30:1. In particular, HPAE-EB at a w/w ratio of 20:1 was able to achieve the highest level of transfection with the CRISPR-C7 plasmid, in line with previous transfections in HEK293 cells. One reason for the lower levels of GFP expression in the presence of more DNA could be due to suboptimal biophysical properties for the particular cell type, such as inefficient DNA release within cells and a limited cell capability to process exogenous DNA. This finding highlights the substantial hurdle associated with translating transfection assessments from an established cell line onto a relevant disease model that is known for being difficult to transfect.

The safety profile of a gene delivery agent can be a significant limiting factor deterring further clinical development. Given the already damaged skin of patients with epidermolysis bullosa and the nature of the disorder, only exemplary viability profiles would be tolerated for a therapeutic use irrespective of overall transfection efficiency. AlamarBlue^TM^ cell metabolic health assay was performed 72 h after transfection for all transfection conditions. Figure 10b shows that all treatment conditions preserved cell viability above 85%, further underlining the well tolerated cytocompatibility of the HPAE-EB polymer. No transfection condition was found to be significantly different from untreated controls, and viabilities were nearly identical between Lipofectamine 3000 and HPAE-EB.

**Fig. 11:**
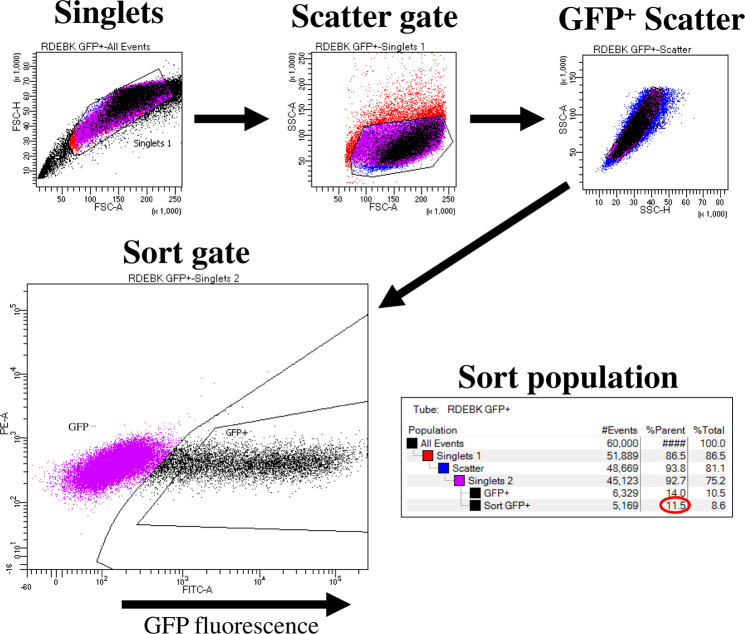
FACS analysis of GFP-positive RDEB keratinocytes. Gating strategy stratified cells into three groups, namely, GFP negative, GFP “dim” and GFP “bright”, based on fluorescence intensity. Optimal transfection conditions using HPAE-EB and CRISPR-C7 plasmid resulted in 11.5% RDEB keratinocytes that were highly fluorescent. Data are representative of three replicates from three independent experiments (*n* = 3).

**Fig. 12:**
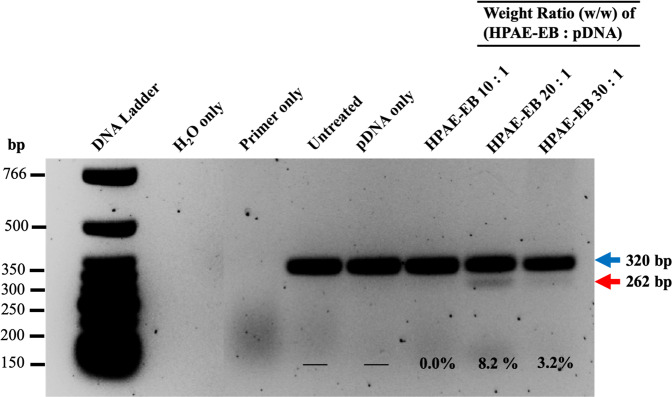
PCR analysis of genomic DNA from RDEB keratinocytes treated under different transfection conditions with 0.5 µg CRISPR-C7 plasmid. Upper arrow of 320 bp band indicates unedited DNA, while lower arrow of 262 bp band corresponds to DNA lacking exon 80. Maximum excision efficiency of 8.2% was estimated by densitometry analysis. Data are representative of three independent experiments (*n* = 3).

Although the transfection efficiency of the RDEB keratinocytes under the optimal conditions identified (20:1 polymer:DNA ratio and 0.5 μg DNA per well) was visibly lower than what was seen using HEK293 cells, it was further analysed to discover if sufficient efficiency had been achieved to mediate a visible correction at least at the genomic DNA level for excision of exon 80. For that purpose, FACS was performed on RDEB cells transfected with HPAE-EB using the optimal transfection conditions above, to identify the percentage and intensity of GFP-positive cells in the population. From the sort gate graph, it is clear that there is a broad range of GFP intensities present in the cell population. This would point to a heterogenous distribution of transgene copies taken up by cells, resulting in intercellular variation in levels of GFP fluorescence intensity. A second gate used to display cells that were deemed to be highly transfected indicated that 11.5% of the total parent population consisted of highly transfected cells (Fig. 11). Results here correlate well with other research groups who showed similar transfection efficiencies of ~17% in RDEB keratinocytes transiently transfected with a CRISPR plasmid using another commercial reagent Xfect™ [54]. Based on GFP transfections and cell viability assessments, genomic DNA from RDEB keratinocytes transfected by HPAE-EB using 0.5 µg DNA was investigated for presence of deletion of exon 80 by PCR. Densitometry analysis identified that excision of exon 80 mediated by HPAE-EB in the population of cells was 8.2 and 3.2% for w/w of 20:1 and 30:1, respectively, while 10:1 again showed only one single band at 320 bp (Fig. 12).

**Fig. 13:**
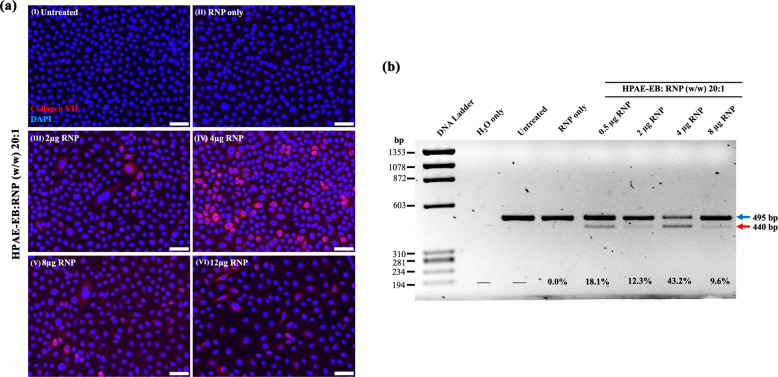
*COL7A1* gene exon 80 excision efficiency in RDEB keratinocytes transfected with CRISPR–Cas9 RNPs complexed to the HPAE-EB polymer. **a** PCR amplification from genomic DNA of a fragment spanning over the sgRNA target sites confirmed removal of exon 80 by the presence of a smaller molecular band (440 bp) in line with the distance between both cut sites (55 bp), marked with a lower arrow (red). Upper arrow (blue) indicates unedited DNA. Densitometry analysis from three independent experiments estimated an exon 80 excision of 43.2% using 4 µg of CRISPR–Cas9 RNP complex at 20:1 w/w ratio with HPAE-EB polymer (*n* = 3). **b** Collagen VII expression (red) was restored in RDEB keratinocytes after CRISPR–Cas9 RNP treatment, assessed by immunofluorescence staining, using DAPI (blue) as a nuclear stain. Magnification at 40×, scale bar 50 µm. Data were collected from two repeats of two independent experiments (*n* = 2).

Due to the low editing efficiency results obtained with the CRISPR–Cas9 plasmid construct in the disease cell model, transfections were performed using HPAE-EB:RNP at w/w ratios of 20:1 across increasing quantities of RNP ranging from 0.5 to 8 µg RNP per well. Using the same overall therapeutic strategy as before, with the plasmid-based approach, again a dual-guide RNA strategy was employed to facilitate excision across exon 80, containing the c.6527insC pathogenic mutation (Fig. 7b). Genomic DNA from all transfection conditions was harvested, and a DNA fragment spanning sgRNA cut sites was PCR amplified and resolved on a 1.5% agarose gel. Untreated cells, and cells treated with dual sgRNA Cas9 RNP with no delivery vector, were used as negative controls not subjected to editing, and these cells exhibited a single 495 bp band as predicted (Fig. 13a). Remarkably, in all treatment conditions using the HPAE-EB polymer to deliver the dual sgRNA Cas9 RNP complexes, a visible lower band is seen at 440 bp, the exact difference between the guide RNA cut sites (55 bp). Densitometry analysis demonstrated that the HPAE-EB treated cells mediated exceptional exon excision efficiencies up to 43.2% in the population of cells. Even the lowest efficiency of exon 80 excision of 9.6%, after transfection with 8 µg RNP, was still greater than the maximum achieved previously using the plasmid-based system earlier in the RDEB keratinocytes. Differences in the delivery of RNP polyplexes (named ribopolyplexes) intracellularly and the release of intact functional RNPs from intracellular compartments could account for the overall varied exon excision efficiency. Moreover, to determine if this editing was translated to partial restoration of the protein production, immunofluorescence staining was performed for collagen VII using a monospecific polyclonal anti-C7 antibody (a generous gift from Dr. A. Nystrom, University of Freiburg). As Fig. 13b details, there was a complete absence of collagen VII expression in untreated RDEB keratinocytes and those treated with RNP only (Fig. 13b, (I) and (II)). In contrast, heterogeneous positive staining was visualised in all cells under HPAE-EB–RNP complex treatments (Fig. 13b, (III)–(VI)), highlighting ribopolyplex-mediated restoration of collagen VII expression in RDEB keratinocytes. Thus, these findings are in line with previous PCR analysis whereby differing overall RNP quantities complexed with HPAE-EB resulted in varied levels of exon 80 removal at the genomic DNA level. As before, cells transfected with HPAE-EB and 4 µg RNP (Fig. 13b (IV)) exhibited the highest collagen VII expression, in line with PCR analysis. As these are preliminary tests, no quantification of the collagen VII expression levels is shown. Further experiments will confirm and provide more details on how well the gene editing achieved translates into increased collagen VII levels.

## Discussion

In this report, we have demonstrated the potential for non-viral polymer delivery for efficient CRISPR–Cas9 genome editing in human cells as a therapeutic approach for achieving targeted genomic deletion of a frequently recurrent mutation site. Previous work by our group highlighted the potential for the HPAE-EB polymer as a broad-spectrum gene delivery agent [2, 3, 12, 13, 26, 27, 30, 31]. Here, we have gone beyond previous assessments by stratifying the transfection process and investigating the HPAE-EB polymers characteristics that enable surpassing the barriers that hinder efficiency at particular stages of the process. Our results show the HPAE-EB polymer can efficiently complex and encapsulate the CRISPR-C7 plasmid. The encapsulation efficiency plateaued around 70% despite the polymer: DNA ratio (w/w) increasing from 10:1 to 30:1. Further increases in the w/w ratio may yield additional encapsulation levels, yet excessive binding affinity could also result in reduced intracellular DNA release and lead to an overall decrease in gene delivery efficiency [8, 9, 55, 56].

The importance that polyplex size, surface charge and buffering capacity play in cell uptake and gene delivery efficiency is well studied, yet not comprehensively understood. Indeed, anionic DNA alone shows negligible uptake through cell membranes, necessitating the use of delivery vectors to enter the cells [57, 58]. Initial polymer delivery vectors such as poly-L-lysine, despite efficiently complexing with DNA, showed poor buffering capacity leading to an inability to escape endosomes and thus low gene delivery efficiency [15, 59]. Here, we demonstrated that HPAE-EB exhibits a high buffering capacity along with forming nanoparticles of sufficient size and charge for traversing the cell membrane. Consistent with our encapsulation and complexation studies, HPAE-EB nucleic acids w/w ratios of 10:1 and above display ideal characteristics to mediate efficient gene delivery in vitro. Nonetheless, further work is required to truly unravel the contributions of polymer backbone design and polyplex characteristics for navigating gene delivery hurdles. In addition, an in-depth analysis of polyplex cell uptake and intracellular trafficking, in particular studying mechanisms of endocytosis and tracking of polyplexes within cellular compartments, would provide valuable insights toward rational design of polymer gene delivery vectors [58, 60–62].

A known limitation in the translation of non-viral gene therapeutics from bench to bedside centres on polyplex scaling and reproducibility [58, 63]. For our initial study, polyplexes were formed by hand-mixing DNA and polymer solutions using a pipette and vortexing. Under such conditions, mixing parameters are nearly impossible to standardise, thus leading to variability in the prepared polyplexes. This was evident in our polyplex size analysis where PDI measurements varied between 0.2 and 0.5, suggesting some polyplex heterogeneity and aggregations were present. To address such limitations, microfluidic mixing systems have been proposed to enable scaling up and biomanufacturing of reproducibly uniform polyplexes to meet clinical requirements [64–66].

Intracellular localisation and production of Cas9 was investigated in transfected cells as it is the functional mediator of gene editing. As Cas9 mediates gene editing via DSBs at target sites, it can be inferred that production of more Cas9, in parallel with the guide RNA, should result in a corresponding increased capacity for gene editing to occur within cells [34, 67, 68]. HPAE-EB transfections indicated a marked localisation of substantial Cas9 expression around the nucleus. While these data provide an initial assessment of the impact of Cas9 localisation on overall gene editing efficiency, full investigation of this aspect would require further studies.

Our therapeutic strategy of targeting and deleting the prevalent mutation site that is exon 80 of COL7A1 gene in RDEB has shown promise in previous publications. Deletion of exon 80 of this gene has successfully restored the reading frame and led to the production of a functional collagen VII protein variant [51, 69]. While these approaches have centred on ex vivo strategies, employing electroporation and viral delivery systems, our approach using HPAE-EB seeks to utilise the in vivo potential of polymer-based delivery systems. Indeed, transfecting and assessing gene editing without enriching the cell population through clonal selection was done to better reflect the polyclonal population of the in vivo environment for clinical application. The fact that genomic deletions of up to 20% could be achieved after a single transfection with HPAE-EB in HEK293 cells, while also maintaining high levels of cell viability, gave cause for optimism. However, when this technology was then assessed in a relevant RDEB cell model (RDEB keratinocytes) which could validate our therapeutic strategy, detectable but very low (maximum of 8.2%) correction in terms of exon 80 excision was achieved. This low level of correction and exon excision would not be predicted to allow for sufficient restoration of type VII collagen. Given that mediating collagen VII restoration is a crucial measure of therapeutic efficacy, the plasmid-based strategy was unable to meet the minimal efficacy criteria to be considered a viable therapeutic option for RDEB at the moment.

However, given the advantages reported for CRISPR–Cas9 delivered as a RNP complex [44, 45], the same dual sgRNA strategy for COL7A1 gene exon 80 excision was used to transfect RDEB keratinocytes to improve editing efficiency in the RDEB model. By using the RNP version of CRISPR–Cas9, much higher levels of COL7A1 gene editing (up to 43.2%) were obtained in a polyclonal population of RDEB keratinocytes and restoration of collagen VII expression was successfully achieved.

It is clear that the CRISPR revolution shows no sign of slowing down with the growth of papers published and the speed with which this gene editing technology is being translated into clinical application [37, 40, 70]. Constant upgrading of guide RNA design and expansion of CRISPR-Cas nucleases are of great benefit, yet enhancing the delivery of therapeutic constructs into target cells and tissues remains paramount for realising the clinical potential of CRISPR technology. Our HPAE-EB polymer highlights the potential for non-viral delivery systems within the gene editing field as a future alternative to viral or electroporation delivery.

In summary, HPAE-EB has ideal biophysical properties to navigate the hurdles for efficient and cytocompatible gene delivery, in this case by targeting the therapeutically relevant exon 80 of the COL7A1 gene, showing its potential for excision of mutation sites as a therapeutic strategy in RDEB using CRISPR–Cas9. The significance of these results is that, by using the HPAE-EB polymer to deliver CRISPR–Cas9 as a RNP, efficient excision of mutant exon 80 and restoration of a functional collagen VII variant can be achieved in a facile one-step process. Moreover, mediating such restoration in a bulk population of cells highlights the potential and promise for further development of this non-viral strategy. Achieving detectable restoration of collagen VII protein after a single treatment in vitro bodes well for downstream optimisation, and for in vivo assessments later, as our data suggest that HPAE-EB may be an invaluable tool for gene editing in future gene therapy applications.

## Supplementary information

Supplementary Information
