# Supplementary material for: Non-viral delivery of CRISPR–Cas9 complexes for targeted gene editing via a polymer delivery system
Source: Gene Ther. 2021 Aug 6;29(3-4):157–70. doi: 10.1038/s41434-021-00282-6 (PMC9013665; doi:10.1038/s41434-021-00282-6)
Supplement: Supplementary file 1 — Supplementary Information [file 41434_2021_282_MOESM1_ESM.pdf]

Supplementary Methods:

Gel Retardation assay:

Polyplexes were prepared as described in Materials and Methods section, to a 10 µl volume. 4 µl of 6X loading dye (Thermo Fisher, Waltham, MA, USA) were added to samples before loading onto a 1% agarose gel (Thermo Fisher) and run for 40 min at 120V. Gels were visualized by SybrSafe DNA stain and imaged using a G-Box transilluminator (Syngene, Bangalore, India). Naked DNA alone was used as the negative control.

PicoGreen DNA Encapsulation Assay:

DNA encapsulation efficiency was assessed using PicoGreen® dsDNA Reagent (Thermo Fisher). Polyplexes were prepared as described in Materials and Methods section, added to a black 96-well plate along with 10 µl of PicoGreen® working solution and incubated for 5 min protected from light. 200 µl of serum free Dulbecco's Modified Eagle Medium (DMEM) 6429 (Sigma Aldrich, St. Louis, MO, USA) were added to wells before fluorescence (F) was read on a SpectraMax M3 multi-plate reader (Molecular Devices, San Jose, CA, USA). DNA encapsulation efficiency (relative fluorescence (RF)) was calculated by:

$RF = (F_{DNA} - F_{sample}) / (F_{DNA} - F_{blank}) \times 100\%$. Wells containing picogreen solution only, diluted in media (F_{blank}) were subtracted as background. F_{DNA} was the fluorescence measurement of free DNA without polymer and F_{sample} was the fluorescence of a given polymer:DNA solution.

Buffering Capacity

HPAE-EB polymer (100 µg/µl, in Dimethyl sulfoxide (DMSO, Sigma Aldrich) was diluted in water to a concentration of 2 mg/ml (total 4 mg in 2 ml) and pH adjusted to 10 using 0.1 M NaOH (Sigma Aldrich). pH was reduced to 3, by adding 20 µl aliquots of 0.1 M HCl (Sigma Aldrich). Branched polyethylenimine (PEI) (25kDa) (Sigma Aldrich) was used as the standard for comparison while 100 mM NaCl was used as the negative control.

Zeta potential and Zetasizer Measurements

Poylplex size, polydispersity index (PDI) and surface charge, were evaluated using a Malvern Zetasizer Nano ZS (Malvern Instrument) equipped with a scattering angle of 173°. Zeta potential samples were measured using a Malvern folded capillary cell (DTS1070), while polyplex size measurements were performed in a clear plastic disposable cuvette. Polyplexes were prepared as described in Materials and Methods section. All samples were further diluted with 980 µl of molecular water for measurement in appropriate cuvette.

Cell Viability Calculations

Untreated cells were used to normalize fluorescence values and plotted as 100% viable. Wells containing alamarBlue™ reagent only were subtracted as background prior to obtaining the % of cell viability, calculated as follows: ($Absorbance_{\mathbf{treatment}}$ /$Absorbance_{\mathbf{untreated}}$) x100%.

Immunocytochemistry

Cells were seeded in 24-well plates containing 12 mm coverslips. These coverslips were washed three times in ice cold phosphate buffer saline (PBS, Thermo Fisher, St. Louis, MO, USA) and then fixed with ice cold acetone:methanol (1:1, Sigma Aldrich) for 20 min at -20 °C. Samples underwent three washes with PBS, then blocking in 3% bovine serum albumin (BSA, Sigma Aldrich) for 20 min at RT and incubated overnight at 4 °C with primary antibody. For probing Cas9, the rabbit monoclonal (ab203933) (Abcam, Cambridge, UK) antibody was used at 1:500 dilution in 0.1% BSA in PBS solution. For collagen VII, a monospecific polyclonal anti-C7 antibody (a generous gift from Dr. A Nystrom, University of Freiburg) was used at 1:5000 dilution in 0.1% BSA in PBS solution as well. Next day, coverslips were incubated for 1 hr in darkness at RT with AlexaFluor 568 nm secondary antibody (A-11031) (Thermo Fisher) at 1:2000 dilution in 0.1% BSA in PBS. Then, coverslips were mounted on microscope slides with Fluoroshield (Abcam) mounting medium and imaged using an Olympus IX83 microscope (Olympus, Tokyo, Japan).

Western Blotting

Cell lysates were obtained by directly adding 100 µl radioimmunoprecipitation assay (RIPA) buffer containing protease cocktail inhibitor (Sigma Aldrich) into well culture plates and collected in 1.5 mL eppendorfs. Lysates were centrifuged for 30 min at 13,000 rpm at 4 °C to pellet cell debris. Protein sample concentrations was determined by Bradford reagent following manufacturer guidelines and samples were boiled for 5 min at 95 °C in Laemmli buffer (4% SDS, 20% glycerol, 125 mM Tris-HCL, 10% β-mercaptoethanol [pH 6.8]) before loading onto 4-20% Mini-PROTEAN TGX gels (Bio-rad, Hercules, CA, USA). Following electrophoresis, samples were electro-transferred onto a nitrocellulose membrane for 2 hrs at 300 mA. Membranes were then blocked on membrane blocking solution (MBS) (Sigma Aldrich) for 1 hr at RT before incubating overnight at 4 °C with target primary antibodies. Rabbit monoclonal (ab203933) antibody was again used, at a dilution of 1:5000. Next day, membranes were incubated with the secondary antibody, conjugated to HRP (anti-rabbit IgG) (ab6721) (Abcam) for 2 hr at 1:2000 dilution at RT and visualized using Pierce ECL western blotting substrate. In the case of the loading control protein GAPDH, a mouse monoclonal primary antibody was used (G8795) (Sigma) at 1:5000 dilution, and a goat anti-mouse (IgG) antibody conjugated to HRP was employed as secondary antibody (ab205719) (Abcam) at a dilution of 1:5000 as well.

PCR Programme Template

PCR program was as follows: 96 °C for 3 min; 5 cycles of 94 °C for 30 s, 68 °C for 30 s, and 72 °C for 45s, decreasing annealing temperature 1 °C every cycle; followed by 30 cycles of 94 °C for 30 s, 63 °C for 30 s, and 72 °C for 45 s; then 72 °C for 7 min.

Supplementary Tables:

PCR Primer	Primer Sequence	%GC Content	TM (^{O}C)
C7-P1	TCTGTGTGTGGTTGTATGTGGA (22)	45.5	58.4
C7-P2	ACCCCACCAAGGAAACTGA (19)	52.6	56.7

Supplementary Table S1. List of PCR primer sequences.

Indel	Frequency	DNA Sequence
**Unedited**	**81.599 %**	**ATTGAGGCTCATCAGTGCCCTCTCTATGTAGGGTCTGCAGGGTCCAAGAGGCCCCCCTGGCCCAGTGGTGAGTACCCAAGAACCTTCACCTGTCTTGCCCCCATCCTGTG**
**-57 bp**	**15.57 %**	**ATTGAGGCTCATCAGTGCCCTCTCT |- |CCTTCACCTGTCTTGCCCCCATCCTGTG**
**+4bp**	**1.07 %**	**ATTGAGGCTCATCAGTGCCCTCTCTnn|ATGTAGGGTCTGCAGGGTCCAAGAGGCCCCCTGGCCCAGTGGTGAGTACCCAAGAAnn|CCTTCACCTGTCTTGCCCCCATCCTGTG**
**-63 bp**	**0.984 %**	**ATTGAGGCTCATCAGTGCCCT- - - - |- | - - TTCACCTGTCTTGCCCCCATCCTGTG**
**-55 bp**	**0.47 %**	**ATTGAGGCTCATCAGTGCCCTCTCTnn|- |CCTTCACCTGTCTTGCCCCCATCCTGTG**
**-64 bp**	**0.273 %**	**ATTGAGGCTCATCAGTGCCCT- - - - |- | - - - TCACCTGTCTTGCCCCCATCCTGTG**
**-63 bp**	**0.034 %**	**ATTGAGGCTCATCAGTGCCCTC - - - |- | - - - TCACCTGTCTTGCCCCCATCCTGTG**
**Σ Editing**	**18.401 %**	
**Σ△Exon 80**	**17.331 %**	

Supplementary Table S2. Indel spectrum generated by ICE analysis software detailing CRISPR edits. INDELs were grouped according to frequency and corresponding DNA sequence.

(i) 0.5µg DNA

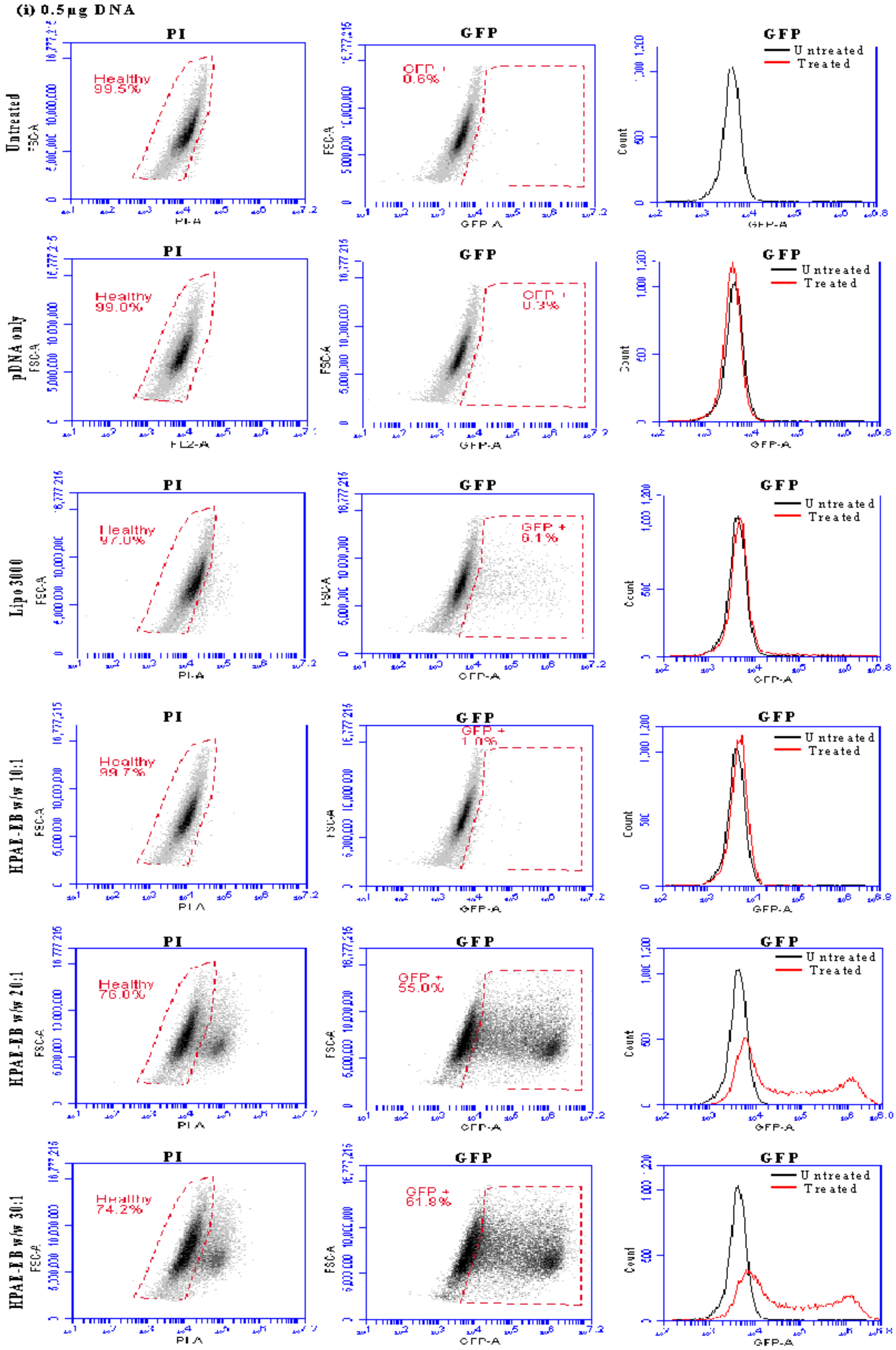

PI
GFP
GFP
Untreated
Treated
Untreated
Healthy 99.5%
GFP + 0.6%
pDNA only
Healthy 99.0%
GFP + 0.3%
Lipo 3000
Healthy 97.0%
GFP + 6.1%
HPAE-EB w/w 10:1
Healthy 99.7%
GFP + 1.0%
HPAE-EB w/w 20:1
Healthy 76.0%
GFP + 55.0%
HPAE-EB w/w 30:1
Healthy 74.2%
GFP + 61.8%
FSC-A
PI-A
GFP-A
Count

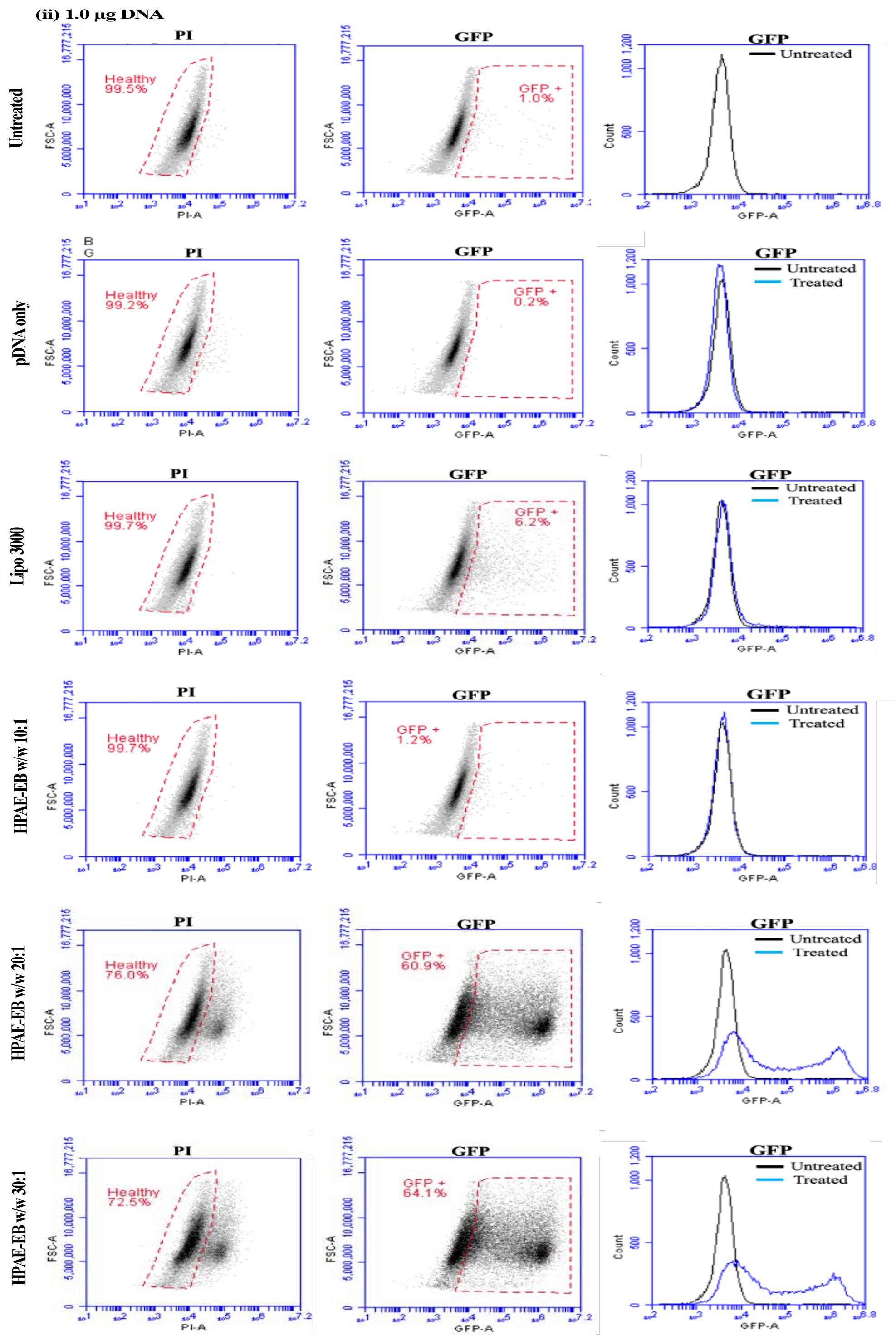

Supplementary Fig. 1. Flow cytometry results of (i) 0.5 µg and (ii) 1 µg CRISPR-C7 plasmid given by viability (1st column), GFP positive population (2nd column) and fluorescence increment (3rd column). Each condition is displayed indicating percentage of healthy cells, percentage of GFP positive cells and a one plot histogram overlaying untreated negative control and treatment sample for GFP fluorescence intensity. HPAE-EB at w/w 10:1

achieved a maximum of 5.8% GFP positive cells. In contrast, HPAE-EB w/w ratios of 20:1 and 30:1 achieved higher levels of GFP positive cells ranging from 55% to 67.3% for different 0.5 and 1 μg of DNA respectively. As is evident from the GFP positive cell population, a substantial shift to the right, indicative with increasing fluorescence is visible. In addition, the one plot histograms overlaying controls and treated samples clearly shows a significant shift in the population fluorescence.
